# Long Wavelength TCF-Based Fluorescent Probe for the Detection of Alkaline Phosphatase in Live Cells

**DOI:** 10.3389/fchem.2019.00255

**Published:** 2019-04-30

**Authors:** Lauren Gwynne, Adam C. Sedgwick, Jordan E. Gardiner, George T. Williams, Gyoungmi Kim, John P. Lowe, Jean-Yves Maillard, A. Toby A. Jenkins, Steven D. Bull, Jonathan L. Sessler, Juyoung Yoon, Tony D. James

**Affiliations:** ^1^Department of Chemistry, University of Bath, Bath, United Kingdom; ^2^Department of Chemistry, University of Texas at Austin, Austin, TX, United States; ^3^Department of Chemistry and Nano Science, Ewha Womans University, Seoul, South Korea; ^4^Cardiff School of Pharmacy and Pharmaceutical Sciences, Cardiff University, Cardiff, United Kingdom

**Keywords:** reaction-based fluorescent probe, alkaline phosphatase, cell imaging, fluorescence, colorimetric

## Abstract

A long wavelength TCF-based fluorescent probe (**TCF-ALP**) was developed for the detection of alkaline phosphatase (ALP). ALP-mediated hydrolysis of the phosphate group of **TCF-ALP** resulted in a significant fluorescence “turn on” (58-fold), which was accompanied by a colorimetric response from yellow to purple. **TCF-ALP** was cell-permeable, which allowed it to be used to image ALP in HeLa cells. Upon addition of bone morphogenic protein 2, **TCF-ALP** proved capable of imaging endogenously stimulated ALP in myogenic murine C2C12 cells. Overall, TCF-ALP offers promise as an effective fluorescent/colorimetric probe for evaluating phosphatase activity in clinical assays or live cell systems.

## Introduction

Alkaline phosphatase (ALP) is an ubiquitous enzyme found in the majority of human tissues, where it catalyses the dephosphorylation of various substrates such as nucleic acids, proteins, and other small molecules (Coleman, 1992; Millán, 2006). ALP also plays an important role in signal transduction and regulation of intracellular processes (cell growth, apoptosis, and signal transduction pathways) (Julien et al., 2011). Abnormal levels of ALP in serum are an indicator of several diseases including bone disease (Garnero and Delmas, 1993), liver dysfunction (Rosen et al., 2016), breast and prostatic cancer (Ritzke et al., 1998; Wymenga et al., 2001), and diabetes (Tibi et al., 1988). As a result, ALP is regarded as a key biomarker in medical diagnosis (Coleman, 1992; Ooi et al., 2007). Therefore, it is important to develop a fast, reliable, and selective detection system for monitoring ALP activity that is amenable to clinical diagnostics.

There have been numerous approaches to determine ALP levels, including colorimetric (Yang et al., 2016; Hu et al., 2017), chemiluminescent (Jiang and Wang, 2012), electrochemical (Zhang L. et al., 2015), surface-enhanced Raman methods (Ruan et al., 2006), and fluorescence (Cao et al., 2016; Fan et al., 2016). Our group has been particularly interested in the development of fluorescent probes for the detection of biologically relevant analytes (Sedgwick et al., 2017a,b, 2018a,b; Wu et al., 2017; Zhang et al., 2019). Fluorescence has many advantages over other methods owing to its simplicity and high sensitivity/selectivity, providing rapid, non-invasive, real-time detection (Wu et al., 2017). Whilst there have been many fluorophores developed for assaying ALP activity such as organic dyes (Zhang H. et al., 2015; Zhao et al., 2017), conjugated polymers (Li et al., 2014), inorganic semiconductor dots (Qian et al., 2015), and noble metal clusters (Sun et al., 2014), most require high probe concentrations and crucially rely on short wavelength emission, thus limiting their applicability in biological systems. Therefore, ALP probes that operate at long wavelengths are required to allow for deeper tissue penetration and to avoid cell-based autofluorescence (Liu et al., 2017; Tan et al., 2017; Zhang et al., 2017).

## Results and Discussion

### Chemistry

**Scheme 1:**
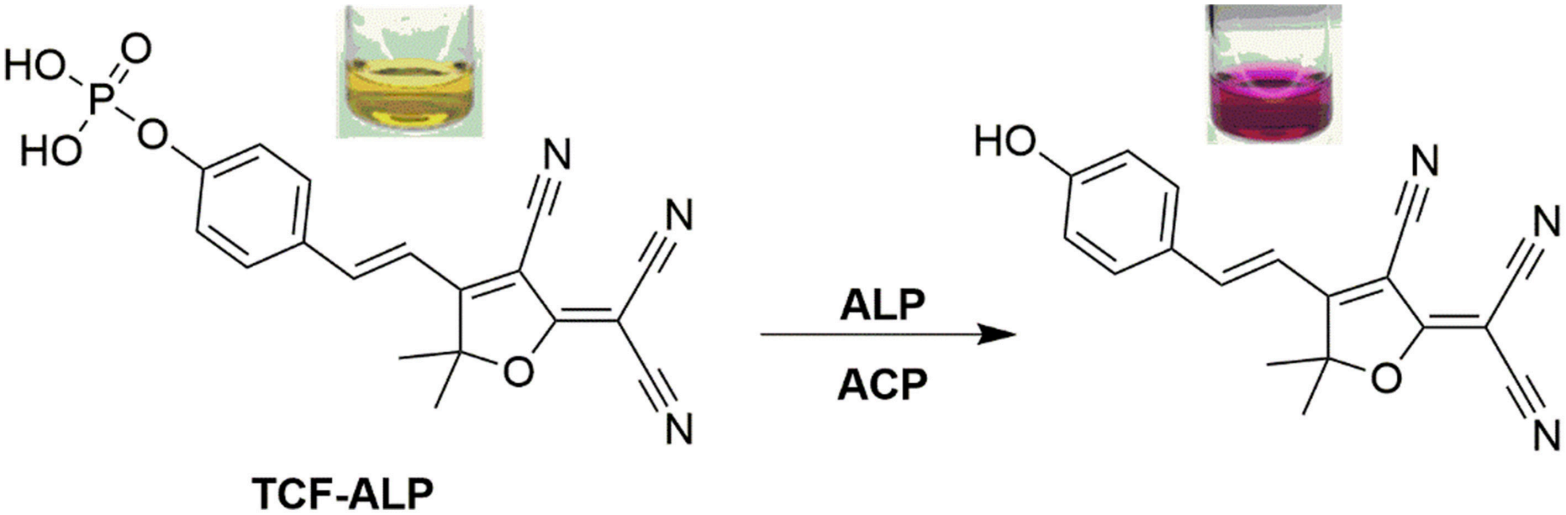
A TCF-based fluorescence probe (**TCF-ALP**) for the detection of alkaline phosphatase.

**Scheme 2:**
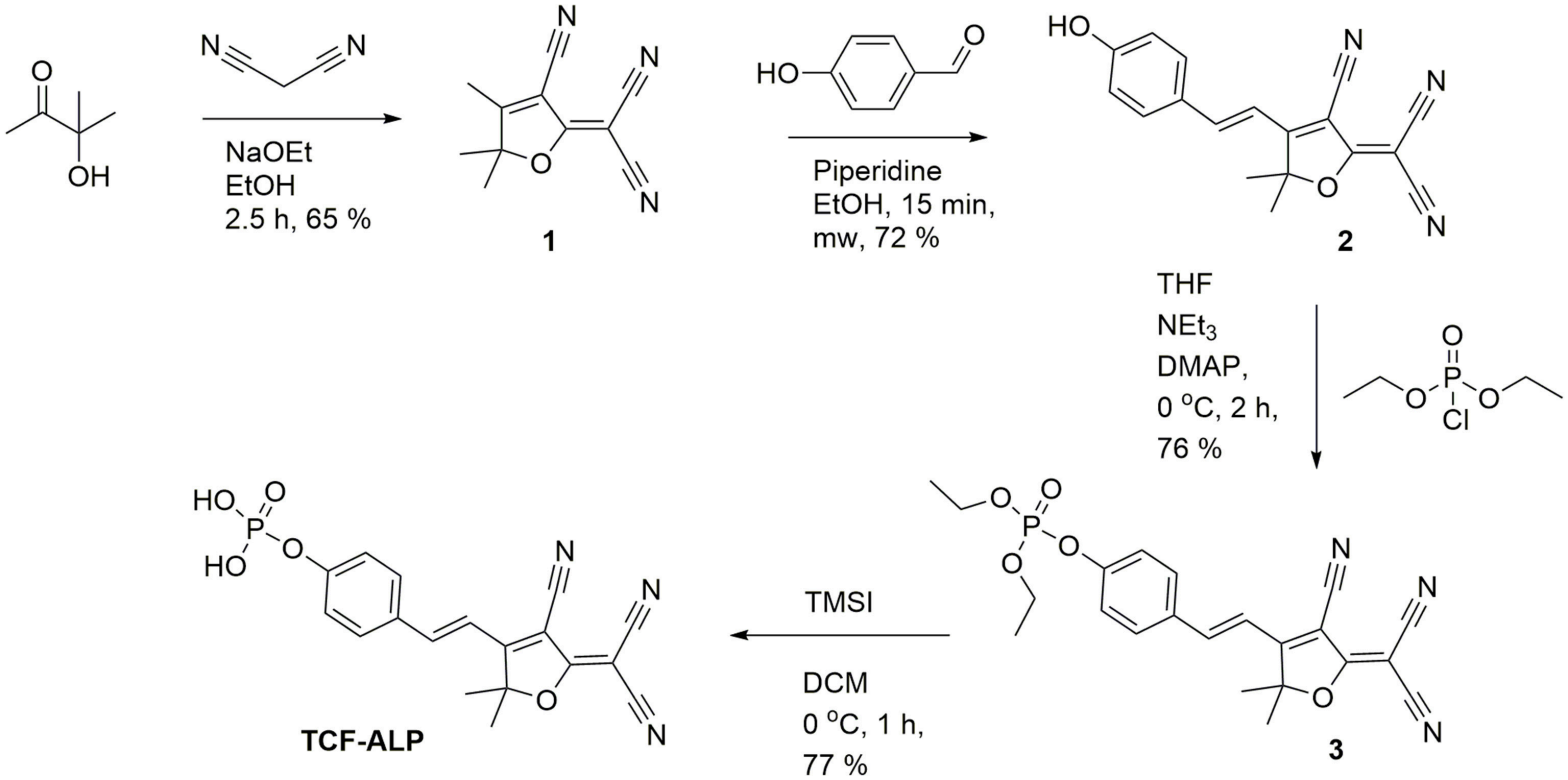
Synthesis of TCF-ALP.

Here we report a TCF-based fluorescent probe that allows for the detection of ALP and/or acid phosphatase (ACP). As shown in Scheme 1, this probe (**TCF-ALP**) is based on the conjugation of 2-dicyanomethylene-3-cyano-4,5,5-trimethyl-2,5-dihydrofuran (**TCF**) to an electron-donating phenol moiety, a phosphorylated phenol; this affords an internal charge transfer (ICT) donor-π-acceptor (D-π-A) system whose fluorescence properties vary dramatically following ALP-mediated phosphate group cleavage (Gopalan et al., 2004; Liao et al., 2006; Bouffard et al., 2008; Lord et al., 2008; Jin et al., 2010; Sedgwick et al., 2017b; Teng et al., 2018). **TCF-ALP** was synthesized in four steps with an overall yield of 27% (Scheme 2). In brief, 3-hydroxy-3-methyl-2-butanone, malononitrile, and NaOEt were heated at reflux in EtOH for 1 h and then cooled. The resultant precipitate **TCF (1)** was then added to a mixture of piperidine (cat.) and 4-hydroxybenzaldehyde in EtOH, which was subsequently heated to 100°C by microwave irradiation to afford intermediate **2** (**TCF-OH**). Intermediate **2** was then treated with diethylchlorophosphate, DMAP (cat.) and NEt_3_ in THF to give the phosphonate ester **3**. Hydrolysis using trimethylsilyl iodide in dichloromethane (DCM) afforded **TCF-ALP** as a crystalline solid (After trituration with Et_2_O).

### Spectroscopic Studies of TCF-ALP

UV-Vis and fluorescence spectroscopic titrations of **TCF-ALP** were performed in 50 mM Tris-HCl buffer in the absence and presence of ALP from porcine kidney. In the absence of ALP, **TCF-ALP** was found to have no UV absorption features above ~550 nm; however, upon addition of ALP a bathochromic shift in the UV absorption maximum was observed (from 440 to 580 nm), which was accompanied by a change in color from yellow to purple (Figure S1). ALP-mediated hydrolysis of **TCF-ALP** to form the highly fluorescent phenol (**2**), was confirmed by ^31^P NMR studies and HRMS (see Figures S1–S4). The effect of pH on the rate of ALP mediated hydrolysis of **TCF-ALP** was evaluated. It was found that incubation with 0.8 U/mL of ALP at pH 9.2 resulted in the largest fluorescence response (Figure S5). Consequently, all *in vitro* experiments to determine ALP activity were carried out in 50 mM Tris-HCl buffer at pH 9.2.

**Figure 1:**
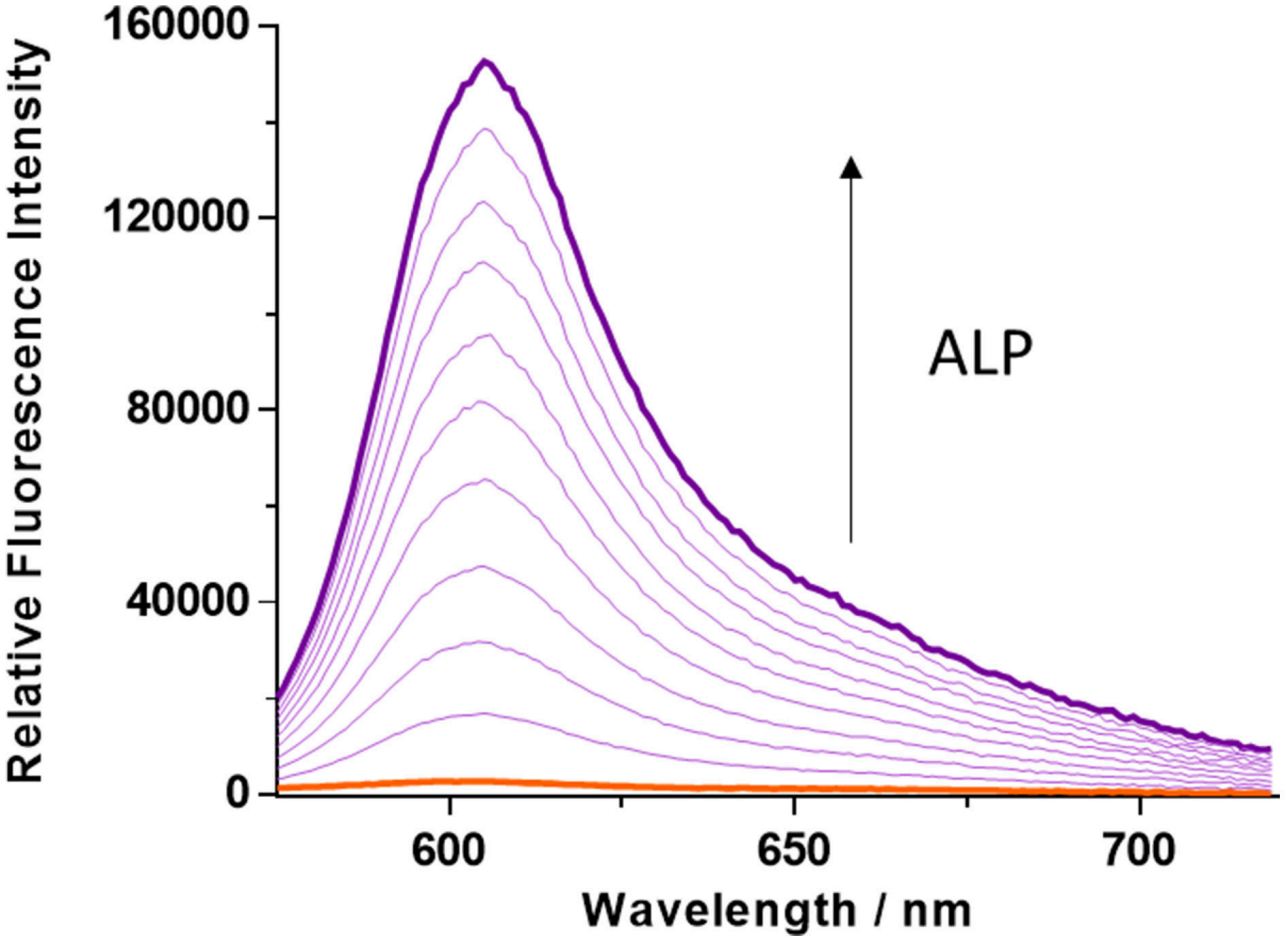
Fluorescence spectra of **TCF-ALP** (10 μM) produced via the addition of alkaline phosphatase (ALP; 0–0.2 U/mL) in 50 mM Tris-HCl buffer, pH = 9.2 at 25°C. λ_ex_ = 542 (bandwidth 15) nm. All measurements were made 15 min after the addition of ALP.

The kinetics of ALP toward **TCF-ALP** were determined via fluorescence spectroscopy (Figures S6, S7), with the resultant fluorescence data analyzed using the Michaelis-Menten equation (Figure S8). This revealed a K_m_ of 35.81 ± 2.63 μM and a V_max_ of 3029 ± 157.3 min^−1^ for hydrolysis of **TCF-ALP** by ALP at pH 9.2 (see Supplementary Material for details). **TCF-ALP** was then incubated with various concentrations of ALP (0.0–0.2 U/mL) for 15 min to evaluate its ability to monitor ALP activity. As shown in Figure 1, a significant fluorescence response was observed in the presence of ALP (58-fold) with a limit of detection (LOD) calculated as 0.12 mU/mL (Figure S9). This sensitivity is comparable to other fluorescent probes found in the literature (Table S3). Although serum alkaline phosphatase levels vary with age in normal individuals (Kattwinkel et al., 1973), it is widely accepted that serum ALP levels in healthy adults lies between 39 and 117 U/mL (Saif et al., 2005; Sahran et al., 2018). This suggests that **TCF-ALP** is capable of detecting ALP in human serum, and therefore could be used in clinical assays.

Inhibition studies were carried out in the presence of sodium orthovanadate (Na_3_VO_4_), which is known to be a strong inhibitor of ALP activity. Addition of Na_3_VO_4_ resulted in a decrease in the fluorescence response in the **TCF-ALP** hydrolysis assay (see Figure S10) (Swarup et al., 1982). These inhibition studies enabled an IC_50_ of 6.23 μM to be calculated (Figure S11), which is similar in value to other ALP substrates that have been reported in the literature (Zhang H. et al., 2015; Tan et al., 2017).

**Figure 2:**
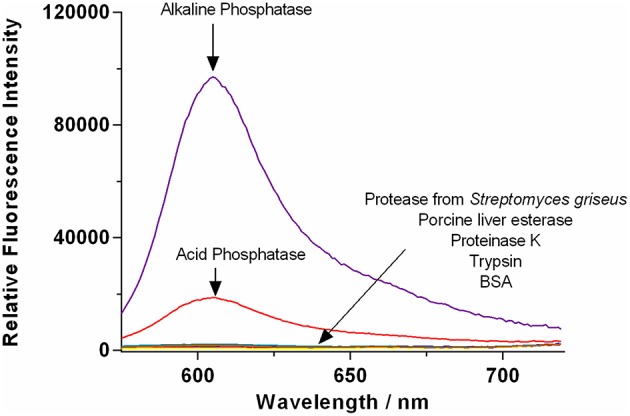
Fluorescence spectra of **TCF-ALP** (10 μM) recorded in the presence of trypsin (0.8 BAEE U/mL), porcine liver esterase, protease from *Streptomyces griseus*, proteinase K, bovine serum albumin (0.1 mg/mL), acid phosphatase (50 mM Tris-HCl, pH = 5.0), and alkaline phosphatase (50 mM Tris-HCl, pH = 9.2). All enzymes were standardized to 0.8 U/mL in Tris-HCl buffer pH 7.1 unless otherwise stated. λ_ex_ = 542 (bandwidth 15) nm/ λ_em_ = 606 nm. Fluorescence measurements were made 30 min after adding the enzyme in question.

The selectivity of **TCF-ALP** toward other biologically relevant enzymes (at their optimal pH values) was then determined (Figure 2 and Figure S12), with **TCF-ALP** displaying high substrate selectivity for ALP over other common hydrolytic enzymes (e.g., trypsin, porcine liver esterase) or non-specific binding proteins [e.g., bovine serum albumin (BSA)]. Interestingly, **TCF-ALP** produced a fluorescence response when treated with ACP. The detection of this enzyme is of significance since it is a tumor biomarker for metastatic prostate cancer (Makarov et al., 2009). Normal levels of ACP in serum range from 3.0 to 4.7 U/mL, and elevated ACP levels can be indicative of a variety of other diseases (Bull et al., 2002). Furthermore, **TCF-ALP** proved capable of detecting ACP (25-fold fluorescence enhancement) and ALP (38-fold enhancement) at a physiological pH of 7.1 (Figures S13, S14). Kinetic determination of ALP and ACP toward **TCF-ALP** at pH 7.1 was conducted, and the resultant K_m_ and V_max_ were compared (see Supplementary Material 3.1 and Figures S15–S18). It was found that ALP has a smaller K_m_ value in comparison to ACP (0.38 ± 0.042 μM and 99.22 ± 13.16 μM, respectively) and a lower V_max_ (208 ± 3.81 min^−1^ and 1962 ± 223.6 min^−1^, respectively). Hence, ALP has higher affinity toward **TCF-ALP** compared to ACP, thus **TCF-ALP** is more selective toward ALP at physiological pH.

According to current standards, determination of ALP and ACP is undertaken at the phosphatase's optimum pH. For example, the Centers for Disease Control and Prevention (CDC) procedure for ALP determination is carried out in 2-amino-2-methyl-1-propanol (AMP) buffer at pH 10.3 [Centers For Disease Control Prevention (CDC), 2012]. This is in accordance with other literature sources (Di Lorenzo et al., 1991; Radio et al., 2006; Pandurangan and Kim, 2015; Guo et al., 2018). Likewise, ACP determination is carried out at pH 4–6 (Li et al., 1984; Boivin and Galand, 1986; Myers and Widlanski, 1993). Following these observations, further studies were conducted to determine selectivity at pH 5.0 and 9.2 (Figures S19–S22). Results showed that **TCF-ALP** acts selectivity toward ACP at acidic pH, and ALP at alkaline pH. Therefore, **TCF-ALP** can be used to selectively detect ALP/ACP in clinical assays, or live cell systems (provided the buffer solution is optimal for the phosphatase under study).

### Imaging of ALP in Living Cells

Prior to exploring whether **TCF-ALP** could be used to image ALP activity levels in live cells, the cytotoxicity of **TCF-ALP** was assessed using a MTT assay (Figure S23). Negligible cell toxicity was observed for **TCF-ALP** concentrations between 0 and 5 μM, and cell viability was only slightly reduced (91%) when incubated with 10 μM **TCF-ALP**, indicating good biocompatibility.

**Figure 3:**
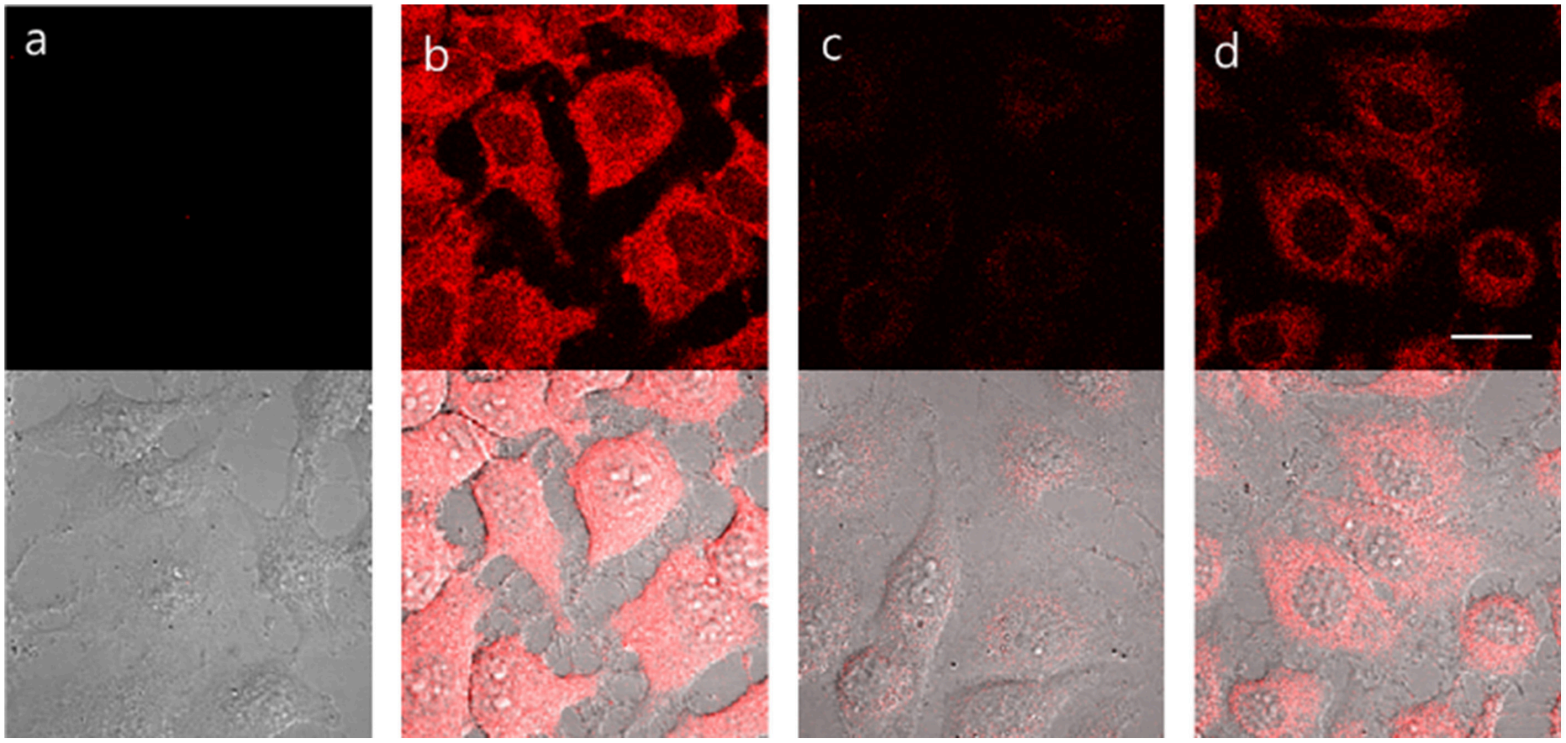
HeLa cells incubated under the following conditions: **(a)** No treatment. **(b) TCF-ALP** (10 μM, 30 min). **(c)** Pre-treated with Na_3_VO_4_ (5 mM, 30 min), followed by the addition of **TCF-ALP** (10 μM, 30 min). **(d)** Pretreated with Na_3_VO_4_ (0.5 mM, 30 min) and **TCF-ALP** (10 μM, 30 min). Cells were washed with DPBS before their fluorescence images were acquired using a confocal microscope. Top half: fluorescence images, bottom half: fluorescence images merged with its corresponding DIC image. Ex. 559 nm/em. 575–675 nm. Scale bar: 20 μm. DIC, differential interference contrast.

**TCF-ALP** proved cell permeable to HeLa cells that express ALP and provided a clear “turn on” response (Figure 3). In contrast, pre-treatment of HeLa cells with Na_3_VO_4_ (5 mM) prior to incubation with **TCF-ALP** resulted in minimal “turn on.” This was taken as evidence that the increase in **TCF-ALP** fluorescence levels seen for HeLa cells in the absence of Na_3_VO_4_ is due to ALP activity. We thus conclude **TCF-ALP** is a probe that allows for the selective cellular imaging of ALP activity.

**Figure 4:**
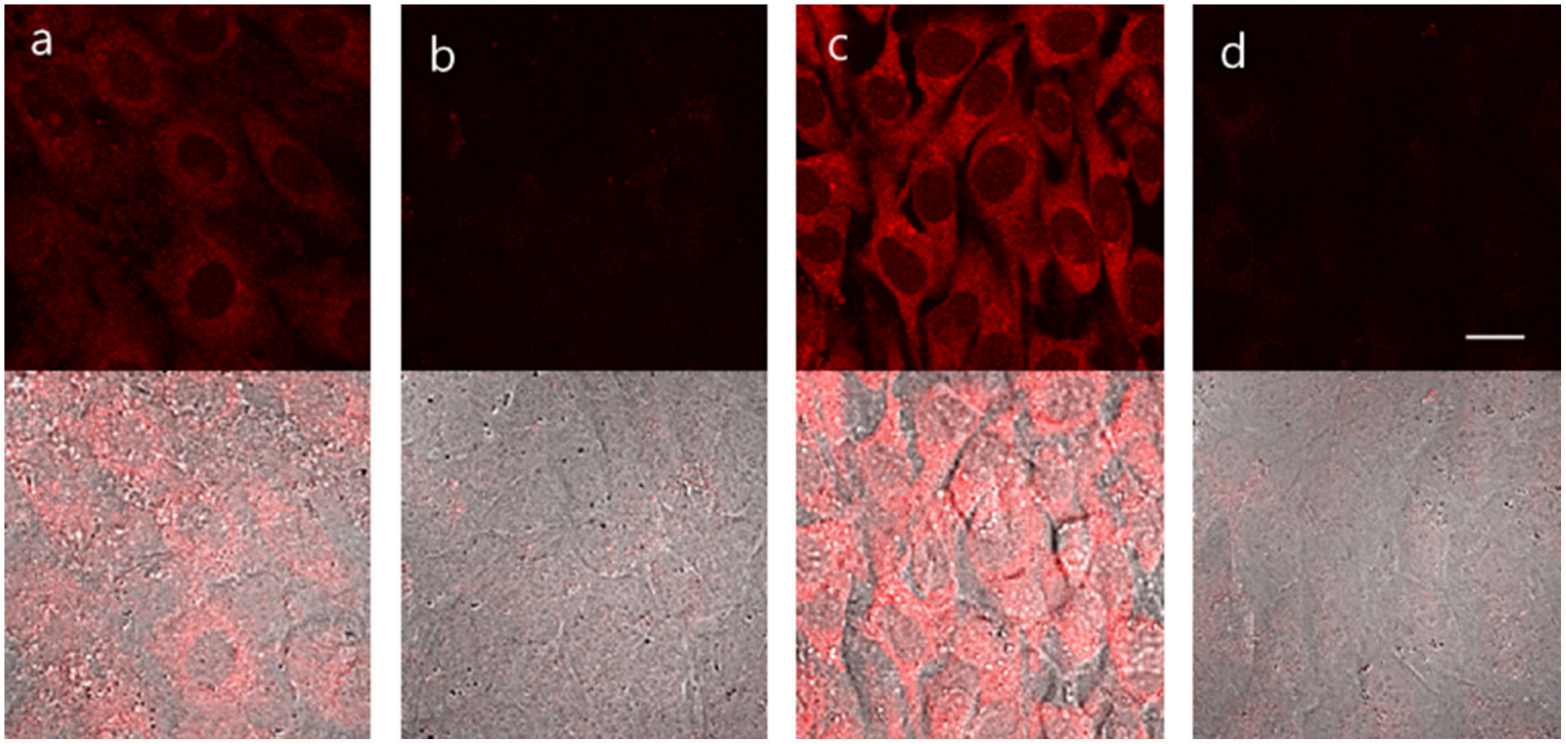
**TCF-ALP** in C2C12 cells. C2C12 cells were treated with 300 ng/mL BMP-2 for 3 days and then pretreated with 5 mM levamisole for 30 min and stained with 10 μM **TCF-ALP** for 30 min. After washing with DPBS, fluorescence images were acquired by confocal microscopy. **(a)** only **TCF-ALP**, **(b)** levamisole + **TCF-ALP**, **(c)** BMP-2 + **TCF-ALP (d)** BMP-2 + levamisole + **TCF-ALP**. Top: fluorescence images, bottom: merged with DIC image. Ex. 559 nm/em. 575–675 nm. Scale bar: 20 μm. DIC, differential interference contrast.

Bone morphogenetic protein 2 (BMP-2) is capable of inducing osteoblast differentiation into a variety of cell types (Guo et al., 2014; Wang et al., 2015) via pathways that result in increased ALP mRNA expression, leading to increased ALP activity (Kim et al., 2004). Treatment of myogenic murine C2C12 cells with **TCF-ALP** resulted in a low fluorescence intensity (low ALP levels) being observed (Figure 4); however, pre-treatment of these cells with BMP-2 (300 ng/mL, 3 days) resulted in a significant increase in **TCF-ALP**-derived fluorescence intensity (high ALP levels). Once again, pre-incubation with Na_3_VO_4_ (5 mM) led to no fluorescence response being observed in the cells treated with **TCF-ALP** (with or without BMP-2). This provided support for the notion that **TCF-ALP** is capable of imaging endogenous ALP activity induced by BMP-2.

## Conclusions

In summary, a long wavelength TCF-based fluorescent probe (**TCF-ALP**) has been prepared with the goal of detecting ALP activity. ALP Hydrolysis of the phosphate group of **TCF-ALP** resulted in a significant “turn on” fluorescence response (58-fold) within 15 min. These spectroscopic changes were accompanied by a colorimetric change from yellow to purple. This enables **TCF-ALP** to be used as a simple assay for the evaluation of ALP activity. Further analysis revealed that **TCF-ALP** could also be used as a probe for detecting ACP activity. **TCF-ALP** was shown to be cell permeable, enabling its use as a fluorescent probe for monitoring ALP levels in HeLa cells. **TCF-ALP** also proved capable of imaging endogenously stimulated ALP produced in myogenic murine C2C12 cells through the addition of bone morphogenetic protein 2. We thus suggest that **TCF-ALP** offers promise as a tool for measuring ALP and ACP activity levels in clinical assays or in live cell systems.

## Author Contributions

LG and AS carried out synthetic and spectroscopic experiments and co-wrote the manuscript with TJ and JS. JG and GW carried out background experiments. GK carried out cellular imaging experiments. JL carried out the ^31^P NMR titrations. J-YM and AJ are supervisors of LG and GW. SB, JY, JS, and TJ all conceived the idea and helped with the manuscript.

### Conflict of Interest Statement

The authors declare that the research was conducted in the absence of any commercial or financial relationships that could be construed as a potential conflict of interest.
