# Supplementary material for: Long Wavelength TCF-Based Fluorescent Probe for the Detection of Alkaline Phosphatase in Live Cells
Source: Front Chem. 2019 Apr 30;7:255. doi: 10.3389/fchem.2019.00255 (PMC6508040; doi:10.3389/fchem.2019.00255)
Supplement: Supplementary file 1 [file Data_Sheet_1.docx]

Supplementary Information

Long wavelength TCF-based fluorescent probe for the detection of Alkaline Phosphatase in live cells

Lauren Gwynne,^a^ Adam C. Sedgwick,^b^ Jordan E. Gardiner,^a^ George T. Williams,^a^ Gyoungmi Kim,^c^ John. P. Lowe,^a^ Jean-Yves Maillard,^d^ A. Toby A. Jenkins,*^a^ Steven D. Bull,*^a^ Jonathan L. Sessler,^b^* Juyoung Yoon*^c^ and Tony D. James*^a^

^a^Department of Chemistry, University of Bath, Bath, BA2 7AY, U.K.

^b^Department of Chemistry. University of Texas at Austin, 105 E 24th street A5300, Austin, TX 78712-1224 (USA)

^c^Department of Chemistry and Nano Science, Ewha Womans University, Seoul 120-750, Korea.

^d^Cardiff School of Pharmacy and Pharmaceutical sciences, Cardiff University, Cardiff, UK

**Corresponding authors.**

T.D.James@bath.ac.uk

[sessler@cm.utexas.edu](mailto:sessler@cm.utexas.edu)

A.T.A.Jenkins@bath.ac.uk

[jyoon@ewha.ac.k](mailto:jyoon@ewha.ac.k)

[S.D.Bull@bath.ac.uk](mailto:S.D.Bull@bath.ac.uk)

Table of Contents

[1. UV analysis 3](#_Toc5697817)

[2. Confirmation of ALP-mediated hydrolysis 3](#_Toc5697818)

[3. Fluorescence analysis 6](#_Toc5697819)

[4. Methods for cell culture and imaging 18](#_Toc5697820)

[5. Experimental section 20](#_Toc5697821)

[6. NMR Spectra 24](#_Toc5697822)

[7. References 33](#_Toc5697823)

# UV analysis

**
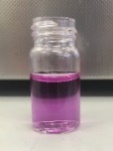

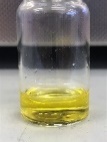


**

Alkaline

phosphatase

**Figure S1:** UV-Vis spectra of **TCF-ALP** (10 µM) recorded with and without 0.8 U/mL of alkaline phosphatase in 50 mM Tris-HCl buffer pH = 9.2.

# Confirmation of ALP-mediated hydrolysis


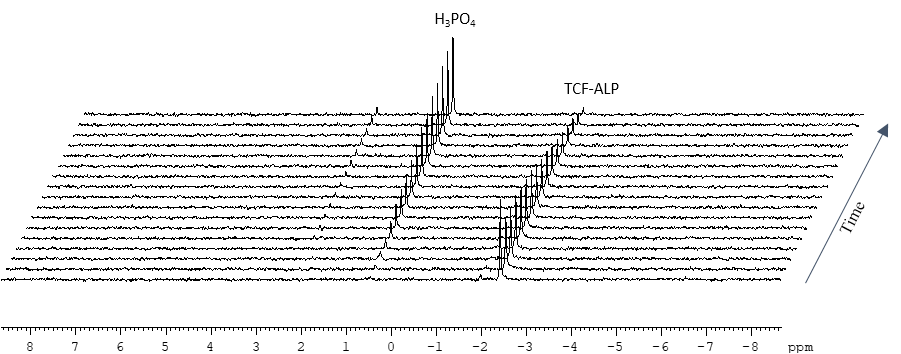


**Figure S2:** ^31^P NMR monitoring of **TCF-ALP** in the presence of ALP in 50 mM Tris-HCl buffer pH = 9.2. Scans were performed at 25 °C every 4 min for 1 h. The spectra show decreasing **TCF-ALP** concentration (~-2.4 ppm) with increasing product formation (H_3_PO_4;_ ~0.5 ppm) over time.


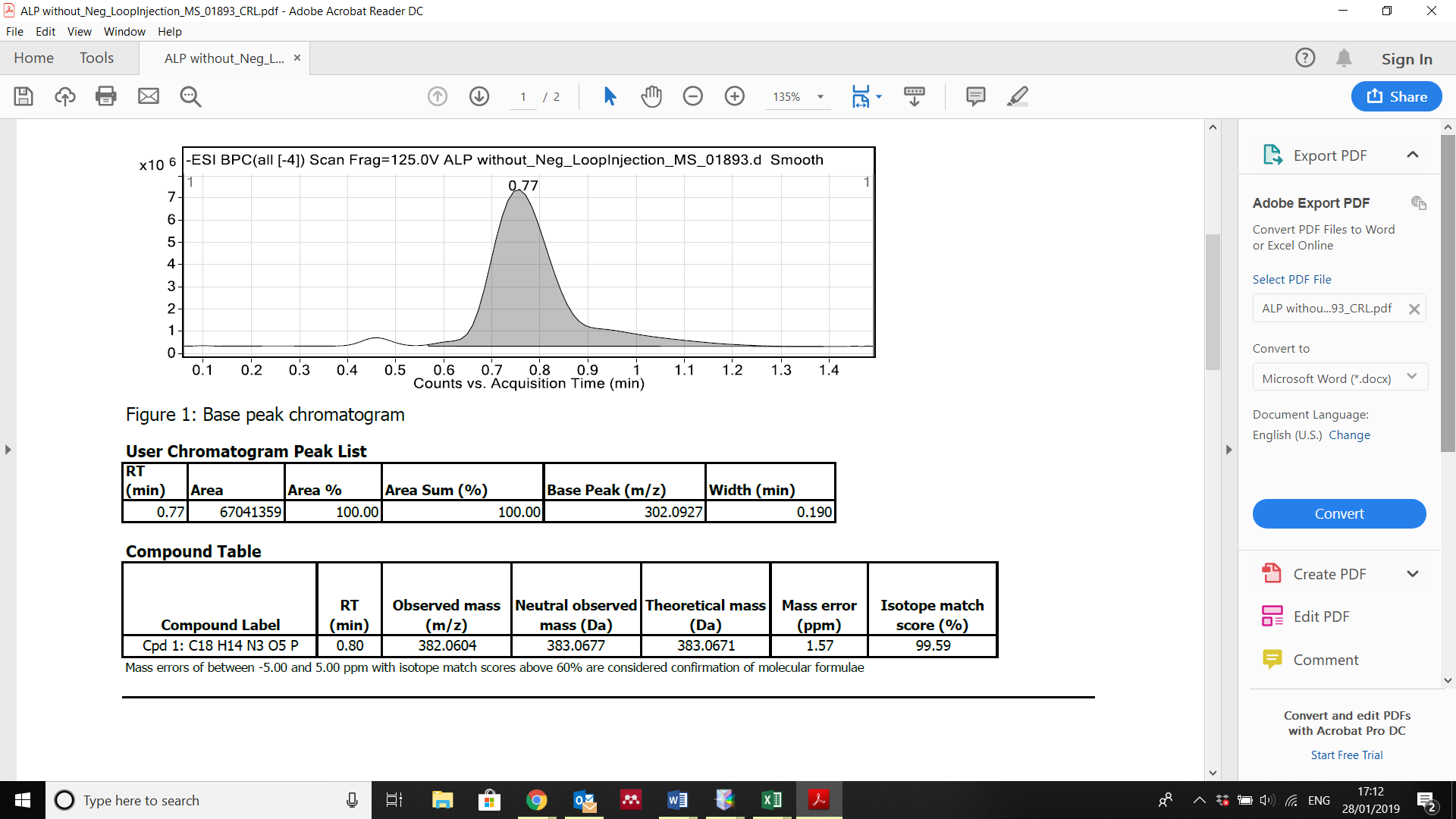


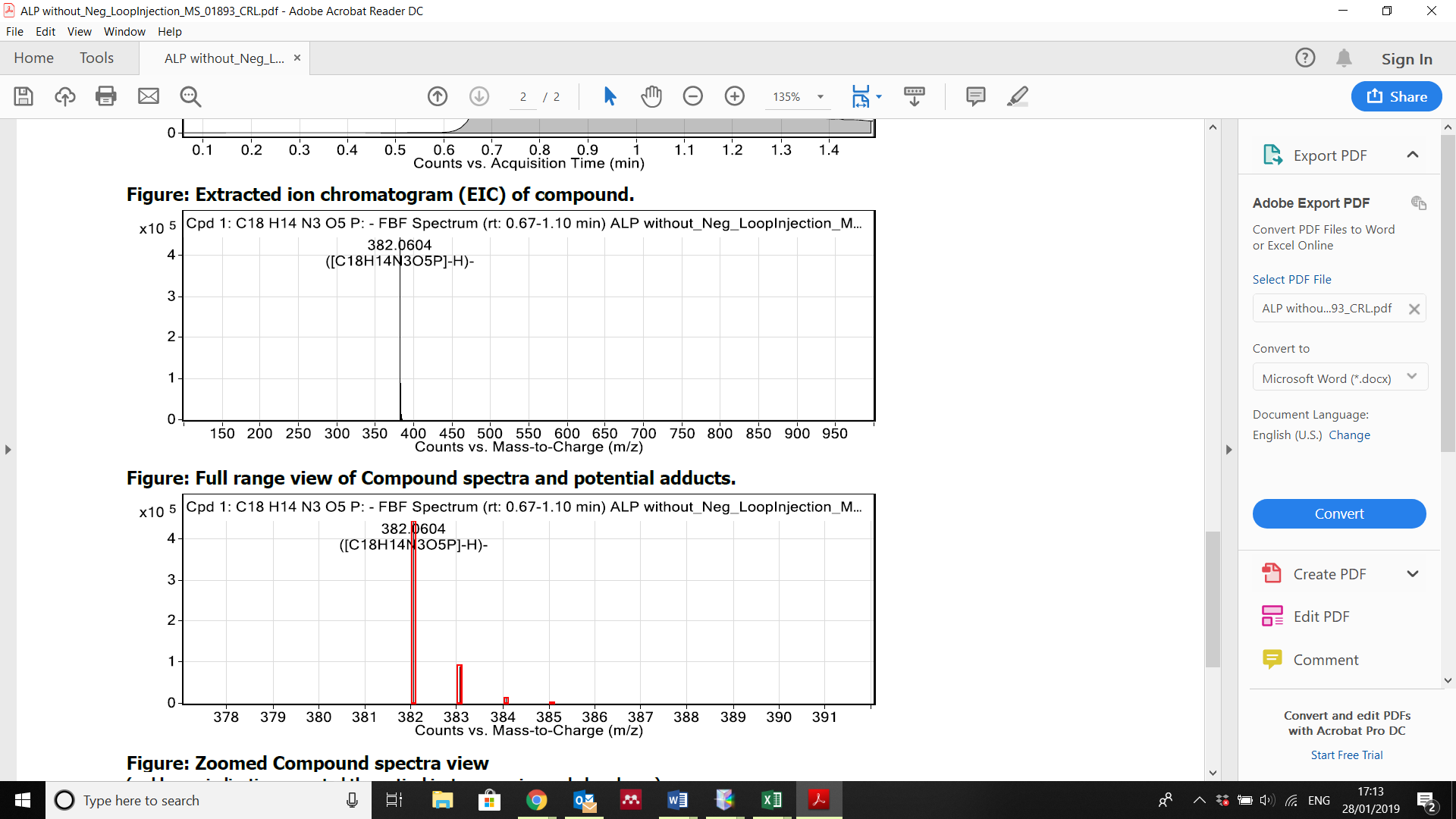


**Figure S3**: HRMS (FTMS-NSI) of **TCF-ALP**: m/z calculated for C_18_H_14_N_3_O_5_P: requires 382.0598 for [M-H]^-^, found 382.0604.


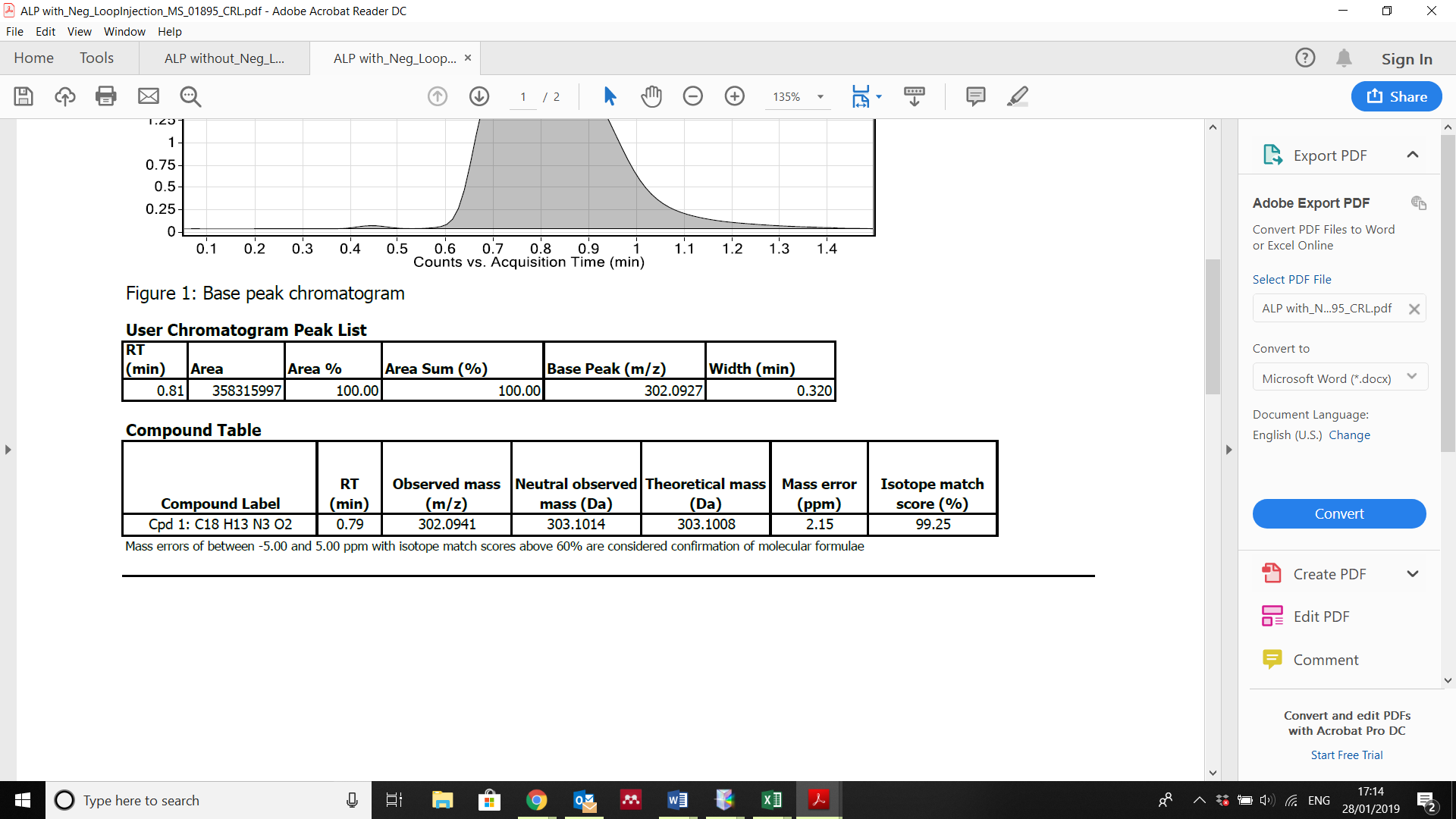


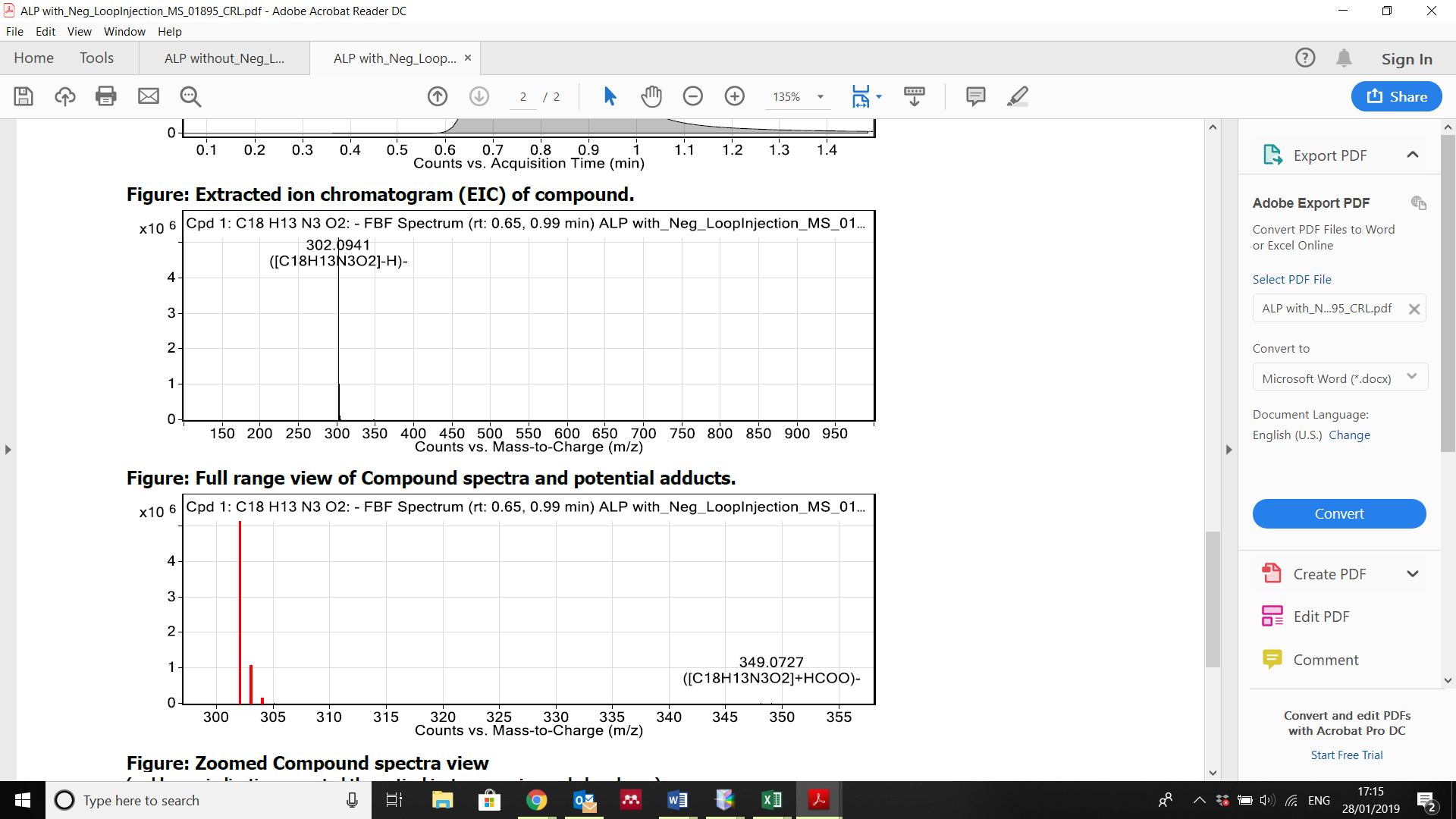


**Figure S4**: HRMS (FTMS-NSI) of **TCF-ALP** with the addition of ALP: m/z calculated for C_18_H_13_N_3_O_2_: requires 302.1081 for [M-H]^-^, found 302.0941.

# Fluorescence analysis

**

**

**Figure S5:** Relative fluorescence intensity of **TCF-ALP** (10 µM) as determined with and without alkaline phosphatase (0.8 U/mL) in 50 mM Tris-HCl buffer at pH 3 – 10. Measurements taken 1 h after incubation at 25 °C. λ_ex_ = 542 (bandwidth 15 nm)/ λ_em_ = 606 nm.. Error bars indicate standard deviation (n = 3).





**Figure S6:** Fluorescence time course seen upon the addition of alkaline phosphatase (0 – 0.8 U/mL) to **TCF-ALP** (10 µM) in 50 mM Tris-HCl buffer pH = 9.2. Measurements for 1 h after incubation at 25 °C. λ_ex_ = 542 (bandwidth 15 nm)/ λ_em_ = 606 (bandwidth 20) nm.

**3.1 Michaelis-Menten kinetics**

The kinetics were analysed using the following expression:

$$\upsilon_{0}=\frac{V_{max}[S]}{K_{m}+[S]}$$

Where V_max_ is the maximum velocity, [S] is the concentration of the **TCF-ALP** substrate, and *K*_m­_ is the Michaelis-Menten constant. As shown in **Figure S7**, the fluorescence emission intensity was enhanced upon increasing probe concentration. Using non-linear regression, V_max_ was calculated to be 3029 ± 157.3 min^-1^ and *K*_m_ was determined to be 35.81 ± 2.63 µM.





**Figure S7:** Time-dependent fluorescence intensity seen for increasing concentrations of **TCF-ALP** (0 – 20 µM) in the presence of 0.2 U/mL of ALP in 50 mM Tris-HCl buffer pH = 9.2 at 25 °C. λ_ex_ = 542 (bandwidth 15 nm)/ λ_em_ = 606 (bandwidth 20) nm.

**Table S1**: Initial rate of enzymatic reaction (υ_0_) versus [**TCF-ALP**]

Slopes ± SD taken from the linear portion of the curves shown in Figure S7 (i.e., over 10 mins). n = 3.

| **Concentration (µM)** | **Slope ± SD** | **R^2^** |
| --- | --- | --- |
| 20 | 1089 ± 31.48 | 0.9771 |
| 10 | 648.2 ± 15.72 | 0.9838 |
| 5.0 | 377.2 ± 6.561 | 0.9916 |
| 2.5 | 205.8 ± 3.336 | 0.9927 |
| 1.0 | 84.72 ± 1.493 | 0.9914 |
| 0.5 | 49.32 ± 1.171 | 0.9845 |





**Figure S8:** Plot of initial velocity (υ_0_) against **TCF-ALP** concentration. Measurements taken at 25 °C. λ_ex_ = 542 (bandwidth 15) nm/ λ_em_ = 606 (bandwidth 20) nm. Error bars indicate standard deviation (n = 3). R^2^ = 0.9985

**3.2 Limit of detection (LOD)**

The limit of detection (LOD) was calculated using the formula shown below:

LOD = 3σ/slope





**Figure S9:** Relative fluorescence intensity seen for **TCF-ALP** (10 µM) upon the addition of alkaline phosphatase (0 – 0.2 U/mL) in 50 mM Tris-HCl buffer, pH = 9.2 at 25 °C. λ_ex_ = 542 (bandwidth 15) nm/ λ_em_ = 606 nm. Error bars indicate the standard deviation (n = 3). The measurements were made 15 min after the addition of alkaline phosphatase.

**Table S2:** Calculation of the LOD

| **Concentration (U/mL)** | **Relative Fluorescence Intensity at 606 nm** | | |
| --- | --- | --- | --- |
| 0.00 | 2628 | 2624 | 2678 |

LOD = (STDEV(2628, 2624, 2678)*3) / slope

= (30.09 * 3) / 755339

= 90.27 / 755339

= 0.00012 U/mL (0.12 mU/mL)

**Table S3**: Fluorescent probes and their corresponding limit of detection (LOD)

| **Fluorescence Mechanism** | **Emission Wavelength (nm)** | **LOD (mU/mL)** | **Incubation time (min)** | **Reference** |
| --- | --- | --- | --- | --- |
| AIE | 495 | 0.0077 | 60 | ^1^ |
| Carbon dots | ~ 445-465 | 0.90 | 15 | ^2^ |
| Carbon dots | 500 | 0.0003 | 20 | ^3^ |
| ESIPT | 400 | 1.3 | 40 | ^4^ |
| FRET | 494/548/624 | 0.06 | 8 | ^5^ |
| FRET/AIE | 570 | 0.2 | 10 | ^6^ |
| ICP nanoparticles | 738 | 3.00 | 20 | ^7^ |
| Quenching | 402 | 0.27 | 10 | ^8^ |
| ICT | 738 | 3.00 | 20 | ^9^ |
| ICT | 700 | 0.07 | 30 | ^10^ |
| ICT | 550/650 | 3.8 | 30 | ^11^ |
| ICT | 606 | 0.12 | 15 | This Work |

**3.3 Inhibition studies**

**

**

**Figure S10:** Inhibition study with **TCF-ALP** (10 µM) in the presence of different concentrations of Na_3_VO_4_ (0 – 2000 µM). Here, 0.8 U/mL of ALP was incubated with Na_3_VO_4_ in 50 mM Tris-HCl buffer pH = 9.2 for 30 min prior to the addition of **TCF-ALP**. The change in fluorescence was monitored for 1 h at 25 °C. λ_ex_ = 542 (bandwidth 15) nm/ λ_em_ = 606 (bandwidth 20) nm.





**Figure S11:** Inhibition study involving different concentrations of Na_3_VO_4_. Fluorescence intensity was recorded 30 min after incubating **TCF-ALP** (10 µM) with 0.8 U/mL ALP in 50 mM Tris-HCl pH = 9.2 at 25 °C. λ_ex_ = 542 (bandwidth 15) nm/ λ_em_ = 606 (bandwidth 20) nm. Error bars indicate the standard deviation (n = 3).

**3.4 Selectivity of TCF-ALP**

**

**

**Figure S12:** Selectivity bar chart of **TCF-ALP** (10 µM) in the presence of: **1.** alkaline phosphatase (50 mM Tris-HCl, pH = 9.2), **2.** acid phosphatase (50 mM Tris-HCl, pH = 5.0), **3.** bovine serum albumin (0.1 mg/mL), **4.** protease from *Streptomyces griseus*, **5.** porcine liver esterase, **6.** proteinase K, **7.** trypsin (0.8 BAEE U/mL). **8-10.** negative controls at pH 5.0, 7.1 and 9.2, respectively. All enzymes were standardised to 0.8 U/mL in Tris-HCl buffer pH 7.1 unless otherwise stated. λ_ex_ = 542 (bandwidth 15 nm)/ λ_em_ = 606 nm. The measurements were made 30 min after enzyme addition. Error bars indicate the standard deviation (n = 3).

**3.5 Selectivity and Kinetics at pH 7.1**





**Figure S13:** Fluorescence spectra of **TCF-ALP** (10 µM) recorded in the presence of acid phosphatase and alkaline phosphatase. All enzymes were standardised to 0.8 U/mL in Tris-HCl buffer pH 7.1. λ_ex_ = 542 (bandwidth 15) nm. The measurements were made 30 min after enzyme addition.





**Figure S14:** Selectivity bar chart showing the relative fluorescence of **TCF-ALP** (10 µM) determined in the presence of: **1.** alkaline phosphatase, **2.** acid phosphatase and **3.** negative control. All enzymes were standardised to 0.8 U/mL in Tris-HCl buffer pH 7.1. λ_ex_ = 542 (bandwidth 15 nm)/ λ_em_ = 606 nm. The measurements were made 30 min after enzyme addition. Error bars indicate standard deviation (n = 3).





**Figure S15:** Time-dependent fluorescence intensity seen for increasing concentrations of **TCF-ALP** (0 – 20 µM) in the presence of 0.2 U/mL of ALP in 50 mM Tris-HCl buffer pH = 7.1 at 25 °C. λ_ex_ = 542 (bandwidth 15) nm/ λ_em_ = 606 (bandwidth 20) nm.

**Table S4**: Initial rate of enzymatic reaction (υ_0_) versus [**TCF-ALP**] using ALP

Slopes ± SD taken from the linear portion of the curves shown in Figure S15 (i.e., over 10 mins). n = 3.

| **Concentration (µM)** | **Slope ± SD** | **R^2^** |
| --- | --- | --- |
| 20 | 211.0 ± 1.241 | 0.9973 |
| 10 | 201.1 ± 1.946 | 0.9927 |
| 5.0 | 194.9 ± 1.847 | 0.9930 |
| 2.5 | 161.5 ± 2.975 | 0.9739 |
| 1.0 | 164.3 ± 3.358 | 0.9848 |
| 0.5 | 116.0 ± 4.161 | 0.9652 |





**Figure S16:** Plot of initial velocity (υ_0_) against **TCF-ALP** concentration. Measurements taken at 25 °C. λ_ex_ = 542 (bandwidth 15) nm/ λ_em_= 606 (bandwidth 20) nm. Error bars indicate standard deviation (n = 3). K_m_ = 0.3760 ± 0.04188 µM, V_max_ = 208.0 ± 3.810 min^-1^. R^2^ = 0.9804.





**Figure S17:** Time-dependent fluorescence intensity seen for increasing concentrations of **TCF-ALP** (0 – 20 µM) in the presence of 0.2 U/mL of ACP in 50 mM Tris-HCl buffer pH = 7.1 at 25 °C. λ_ex_ = 542 (bandwidth 15) nm/ λ_em_ = 606 (bandwidth 20) nm.

**Table S5**: Initial rate of enzymatic reaction (υ_0_) versus [**TCF-ALP**]

Slopes ± SD taken from the linear portion of the curves shown in Figure S17 (i.e., over 10 mins). n = 3.

| **Concentration (µM)** | **Slope ± SD** | **R^2^** |
| --- | --- | --- |
| 20 | 329.7 ± 2.253 | 0.9963 |
| 10 | 176.7 ± 2.074 | 0.9892 |
| 5.0 | 97.87 ± 1.223 | 0.9878 |
| 2.5 | 48.89 ± 0.4965 | 0.9919 |
| 1.0 | 15.04 ± 1.595 | 0.5296 |
| 0.5 | 14.43 ± 1.393 | 0.5759 |





**Figure S18:** Plot of initial velocity (υ_0_) against **TCF-ALP** concentration. Measurements taken at 25 °C. λ_ex_ = 542 (bandwidth 15) nm/ λ_em_= 606 (bandwidth 20) nm. Error bars indicate standard deviation (n = 3). K_m_ = 99.22 ± 13.16 µM, V_max_ = 1962 ± 223.6 min^-1^.R^2^ = 0.9989.

**3.6 Selectivity of TCF-ALP at pH 5.0 and 9.2**





**Figure S19:** Fluorescence spectra of **TCF-ALP** (10 µM) recorded in the presence of acid phosphatase and alkaline phosphatase. All enzymes were standardised to 0.8 U/mL in Tris-HCl buffer pH 5.0. λ_ex_ = 542 (bandwidth 15) nm. The measurements were made 30 min after enzyme addition.





**Figure S20:** Selectivity bar chart showing the relative fluorescence of **TCF-ALP** (10 µM) determined in the presence of: **1.** alkaline phosphatase, **2.** acid phosphatase and **3.** negative control. All enzymes were standardised to 0.8 U/mL in Tris-HCl buffer pH 5.0. λ_ex_ = 542 (bandwidth 15 nm)/ λ_em_ = 606 nm. The measurements were made 30 min after enzyme addition. Error bars indicate standard deviation (n = 3).





**Figure S21:** Fluorescence spectra of **TCF-ALP** (10 µM) recorded in the presence of acid phosphatase and alkaline phosphatase. All enzymes were standardised to 0.8 U/mL in Tris-HCl buffer pH 9.2. λ_ex_ = 542 (bandwidth 15) nm. The measurements were made 30 min after enzyme addition.





**Figure S22:** Selectivity bar chart showing the relative fluorescence of **TCF-ALP** (10 µM) determined in the presence of: **1.** alkaline phosphatase, **2.** acid phosphatase and **3.** negative control. All enzymes were standardised to 0.8 U/mL in Tris-HCl buffer pH 9.2. λ_ex_ = 542 (bandwidth 15) nm/ λ_em_ = 606 nm. The measurements were made 30 min after enzyme addition. Error bars indicate standard deviation (n = 3).

# Methods for cell culture and imaging

**Cell culture**

HeLa cells (human epithelial adenocarcinoma) were purchased from the Korean Cell Line Bank (Seoul, Korea). Cells were cultured in MEM (Eagle's Minimum Essential Medium) supplemented with heat-inactivated 10% fetal bovine serum, 100 U/mL penicillin and 100 U/mL streptomycin. All cells were kept in 5% CO_2_ at 37 ℃.

**Confocal microscopy imaging**

Cells were seeded in a 35-mm glass bottomed dishes at a density of 3 x 10^5^ cells per dish in culture media. After culturing for 24 h, cells were incubated with 10 μM of the probe for 30 min and washed with DPBS. Fluorescence images were recorded by means of confocal laser scanning microscopy (FV1200, Olympus, Japan). To prevent intracellular ALP activity, a 30 min pretreatment with 5 mM levamisole or 0.5 mM Na_3_VO_4_ (ALP inhibitors) was carried out. To acquire the fluorescence image, cells were excited with a 559 nm laser and a 575‑675 nm emission filter was used.

**Cytotoxicity tests**

Cells were seeded in a 96-well plate with culture media. After culturing overnight, cells were incubated with various concentrations of sample for 24 h. To identify cell viability, reagents were removed and 0.5 mg/mL of MTT (Sigma) was added to the cells, which were then incubated for 4 h at 37 ℃ in a CO_2_ incubator. The formazan produced was dissolved in 0.1 mL of dimethylsulfoxide (DMSO) and read at OD 650 nm with a Spectramax Microwell plate reader. Absorbance was determined and the mean cell viability was calculated as a percentage of the mean vehicle control. Results of are the average of 3 independent experiments.

**4.1 Cytotoxicity of TCF-ALP**

**Figure S23**: HeLa cells were incubated with each concentration of **TCF-ALP** for 24 h. After incubation, cells were treated with MTT media and cultured for another 4 h. Absorbance of no treatment were determined as 100 % live, and results are expressed as mean ± standard deviation of three independent experiments

# Experimental section

**2-(3-Cyano-4,5,5-trimethylfuran-2(5H)-ylidene)malononitrile (1)**

NaOEt (0.391 g, 5.75 mmol) was added to a solution of 3-hydroxy-3-methyl-2-butanone (4 mL, 38 mmol) and malonitrile (4.9 g, 74 mmol) in EtOH (10 mL) and stirred for 1.5 h. The reaction mixture was then refluxed for 1 h, which was then cooled to rt. The mixture was cooled and the solid precipitate was filtered to afford the title compound **(1)** as a pale grey solid (4.92 g, 24.70 mmol, 65%); M.p. 204 – 208 ^o^C (decomp). ^1^H NMR (500 MHz, CDCl_3_) δ 2.37 (s, 3 H), 1.64 (s, 6 H); ^13^C NMR (75.5 MHz, CDCl_3_) δ 182.6, 175.2, 111.1, 110.4, 109.0, 104.8, 99.8, 58.5, 24.4, 14.2; IR (thinfilm) ν max (cm^-1^): 2232.78, 2222.00 (CN); HRMS (FTMS-NSI): m/z calculated for C_11_H_9_N_3_O: requires 200.0108 for [M+H]^+^, found 200.0108.

**(*E*)-2-(3-Cyano-4-(4-hydroxystyryl)-5,5-dimethylfuran-2(5H)-ylidene)malononitrile (2)**

Two drops of piperidine were added to a mixture of 4-hydroxybenzaldehyde (0.122 g, 1 mmol) and TCF **(1)** (0.228 g, 1.15 mmol) in EtOH (10 mL). The reaction mixture was heated in the microwave for 15 min at 100 ^o^C and then allowed to cool to rt. The solid precipitate was filtered off to afford the title compound **(2)** as a red solid (0.22 g, 0.72 mmol, 72%) M.p. 202 – 206 ^o^C (decomp). ^1^H NMR (300MHz, DMSO-*d*_6_) δ 7.95 - 7.73 (m, 3 H), 7.01 (d, *J* = 16.2 Hz, 1 H), 6.89 (d, *J* = 8.7 Hz, 2 H), 1.77 (s, 6 H); ^13^C NMR (75.5 MHz, DMSO-*d*_6_) δ 177.6, 176.2, 162.7, 148.7, 132.7, 126.0, 116.8, 113.3, 112.5, 112.0, 111.6, 99.4, 96.9, 53.5, 25.7; I.R (thinfilm) ν max (cm^-1^): 3361.61 (O-H), 2224.73 (CN); HRMS (FTMS-NSI): m/z calculated for C_18_H_13_N_3_O_2_: requires 304.1081 for [M+H]^+^, found 304.1084.

**(E)-4-(2-(4-Cyano-5-(dicyanomethylene)-2,2-dimethyl-2,5-dihydrofuran-3-yl)vinyl)phenyl diethyl phosphate (3)**

Intermediate **2** (0.20 g, 0.66 mmol) was dissolved in a solution containing THF (10 mL) and NEt_3_ (0.3 mL). This was followed by the addition of DMAP (0.050 g). The resulting solution was cooled to 0 ºC and diethylchlorophosphate (0.14 mL, 1 mmol) was added dropwise over the course of 15 min. The reaction mixture was monitored *via* TLC once the starting material was consumed (~ 2 hrs), EtOAc (50 mL) and H_2_O (50 mL) were added to the reaction mixture. The organic layer was washed with H_2_O (2 x 50 mL) and brine (50 mL). It was then dried (MgSO_4_) and concentrated *in vacuo* to afford the crude material. This crude material was purified *via* column chromatography over silica gel EtOAc:petroleum ether (30:70) to afford the title compound (**3**) as an orange solid (0.22 g, 0.50 mmol, 76%). M.p. 224 -226 °C; ^1^H NMR (500 MHz, CDCl_3_) δ 7.70 - 7.61 (m, 3 H), 7.36 (d, *J* = 8.3 Hz, 2 H), 6.97 (d, *J* = 16.6 Hz, 1 H), 4.30 - 4.20 (m, 4 H), 1.80 (s, 6 H), 1.42 - 1.36 (s, 6 H); ^13^C NMR (125.7 MHz, CDCl_3_) δ 175.1, 173.5, 154.2, 154.1, 146.0, 130.7, 130.5, 121.1, 114.7, 111.5, 110.7, 110.1, 100.1, 97.7, 65.0, 65.0, 58.1, 26.4, 16.1; FTIR (thinfilm) ν max (cm^-1^): 2990.16 (O-H), 2224.47 (CN), 2214.60 (CN). HRMS (FTMS-NSI): m/z calculated for C_22_H_22_N_3_O_5_P: requires 438.1224 for [M-H]^-^, found 438.1233.

(**E)-4-(2-(4-Cyano-5-(dicyanomethylene)-2,2-dimethyl-2,5-dihydrofuran-3-yl)vinyl)phenyl phosphate (TCF-ALP)**

A solution of **3** (0.15 g, 0.34 mmol) in DCM (5 mL) was cooled to 0 ^o^C before the dropwise addition of TMSI (0.1 mL, 0.68 mmol). The reaction mixture was stirred for 1 h before the solvent was removed *in vacuo* to afford the crude solid, which was purified *via* trituration (diethyl ether) to afford an orange solid (0.10 g, 0.26 mmol, 77 %). M.p. 239 – 242 °C; ^1^H NMR (500 MHz, DMSO-d6) δ 7.99 - 7.84 (m, 3 H), 7.30 (d, *J* = 8.3 Hz, 2 H), 7.16 (d, *J* = 16.6 Hz, 1 H), 1.79 (s, 6 H); ^13^C NMR (125. 7 MHz, DMSO-*d*_6_) δ 177.6, 175.7, 155.3, 155.2, 147.1, 131.7, 130.5, 121.1, 121.0, 115.0, 113.1, 112.3, 111.3, 99.8, 99.4, 54.7, 25.6; ^31^P NMR (202.4 MHz, DMSO-*d*_6_) δ -.6.70; FTIR (thinfilm) ν max (cm^-1^): 2980.15 (O-H), 2235.84 (CN). HRMS (FTMS-NSI): m/z calculated for C_18_H_14_N_3_O_5_P: requires 382.0598 for [M-H]^-^, found 382.0596.

# NMR Spectra

**2-(3-Cyano-4,5,5-trimethylfuran-2(5H)-ylidene)malononitrile (1)** (500 MHz, CDCl_3_)

**2-(3-Cyano-4,5,5-trimethylfuran-2(5H)-ylidene)malononitrile (1)** (75.5 MHz, CDCl_3_)

**(E)-2-(3-Cyano-4-(4-hydroxystyryl)-5,5-dimethylfuran-2(5H)-ylidene)malononitrile (2)** (300 MHz, CDCl_3_)

**(E)-2-(3-Cyano-4-(4-hydroxystyryl)-5,5-dimethylfuran-2(5H)-ylidene)malononitrile (2)** (75.5 MHz, CDCl_3_)

**(E)-4-(2-(4-Cyano-5-(dicyanomethylene)-2,2-dimethyl-2,5-dihydrofuran-3-yl)vinyl)phenyl diethyl phosphate (3)** (500 MHz, CDCl_3_)

**(E)-4-(2-(4-Cyano-5-(dicyanomethylene)-2,2-dimethyl-2,5-dihydrofuran-3-yl)vinyl)phenyl diethyl phosphate (3)** (125.7 MHz, CDCl_3_)

**(E)-4-(2-(4-Cyano-5-(dicyanomethylene)-2,2-dimethyl-2,5-dihydrofuran-3-yl)vinyl)phenyl phosphate (TCF-ALP)** (500 MHz, DMSO-d6)

**(E)-4-(2-(4-Cyano-5-(dicyanomethylene)-2,2-dimethyl-2,5-dihydrofuran-3-yl)vinyl)phenyl phosphate (TCF-ALP)** (125.7 MHz, DMSO-*d*_6_)

**^31^P NMR (E)-4-(2-(4-Cyano-5-(dicyanomethylene)-2,2-dimethyl-2,5-dihydrofuran-3-yl)vinyl)phenyl phosphate (TCF-ALP)** (202.4 MHz, DMSO-*d*_6_)

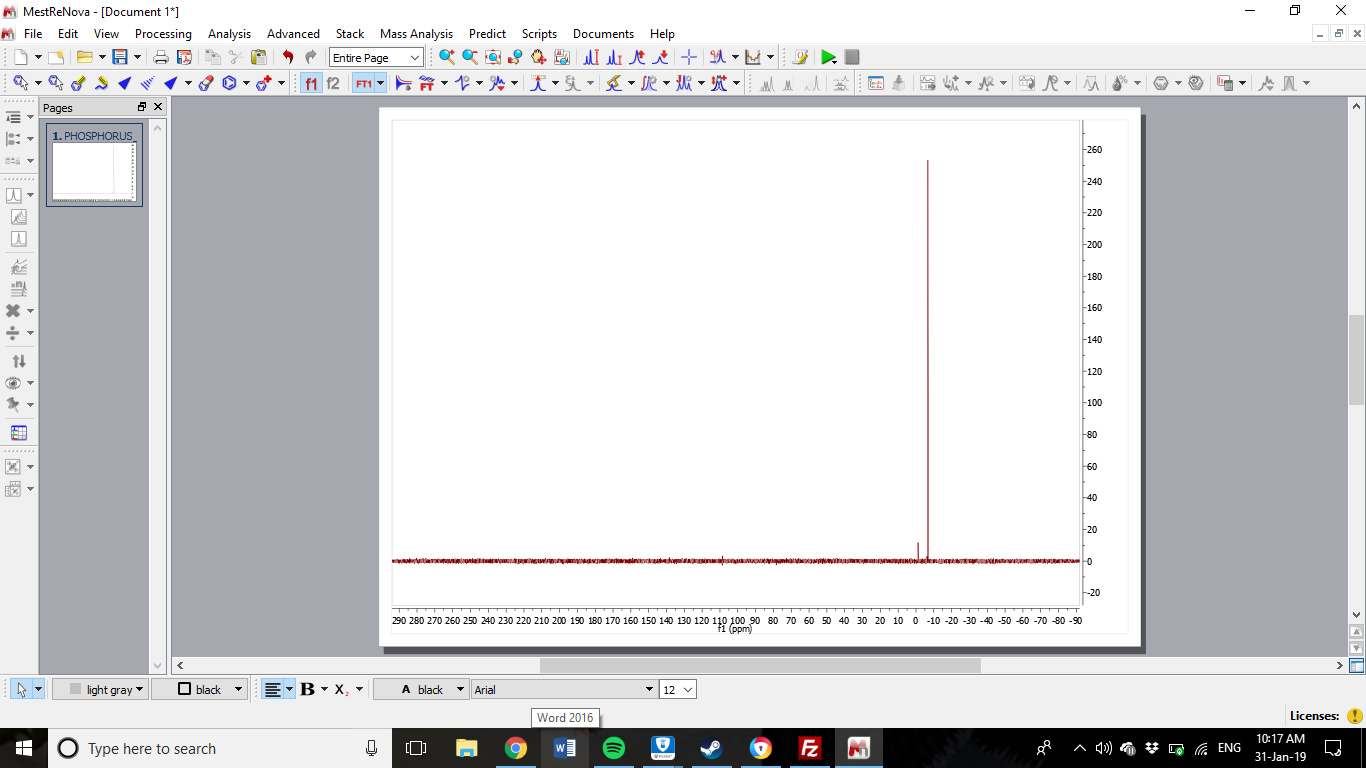


# References

1. W. Zhang, H. Yang, N. Li and N. Zhao, *RSC Advances*, 2018, **8**, 14995-15000.

2. C. Tang, Z. Qian, Y. Huang, J. Xu, H. Ao, M. Zhao, J. Zhou, J. Chen and H. Feng, *Biosensors and Bioelectronics*, 2016, **83**, 274-280.

3. M. Mao, T. Tian, Y. He, Y. Ge, J. Zhou and G. Song, *Microchimica Acta*, 2018, **185**, 17.

4. C. Fan, S. Luo and H. Qi, *Luminescence*, 2016, **31**, 423-427.

5. A.-Z. Xu, L. Zhang, H.-H. Zeng, R.-P. Liang and J.-D. Qiu, *Microchimica Acta*, 2018, **185**, 288.

6. X. Han, M. Han, L. Ma, F. Qu, R.-M. Kong and F. Qu, *Talanta*, 2019, **194**, 55-62.

7. J. Ou-Yang, C.-Y. Li, Y.-F. Li, B. Yang and S.-J. Li, *Sensors and Actuators B: Chemical*, 2018, **255**, 3355-3363.

8. Y. Mei, Q. Hu, B. Zhou, Y. Zhang, M. He, T. Xu, F. Li and J. Kong, *Talanta*, 2018, **176**, 52-58.

9. S.-J. Li, C.-Y. Li, Y.-F. Li, J. Fei, P. Wu, B. Yang, J. Ou-Yang and S.-X. Nie, *Analytical chemistry*, 2017, **89**, 6854-6860.

10. Y. Tan, L. Zhang, K. H. Man, R. Peltier, G. Chen, H. Zhang, L. Zhou, F. Wang, D. Ho and S. Q. Yao, *ACS applied materials & interfaces*, 2017, **9**, 6796-6803.

11. Z. Lu, J. Wu, W. Liu, G. Zhang and P. Wang, *RSC Advances*, 2016, **6**, 32046-32051.
